# Centenary Progress on Orchidaceae Research: A Bibliometric Analysis

**DOI:** 10.3390/genes16030336

**Published:** 2025-03-13

**Authors:** Yonglu Wei, Jie Li, Jianpeng Jin, Jie Gao, Qi Xie, Chuqiao Lu, Genfa Zhu, Fengxi Yang

**Affiliations:** 1 Guangdong Key Laboratory of Ornamental Plant Germplasm Innovation and Utilization, Environmental Horticulture Research Institute, Guangdong Academy of Agricultural Sciences, Guangzhou 510640, China; weiyonglu@gdaas.cn (Y.W.);; 2 School of Landscape Architecture, Beijing Forestry University, Beijing 100083, China

**Keywords:** Orchidaceae, bibliometric analysis, web of science, co-word analysis, phylogenetics

## Abstract

Background: Research on orchids has experienced substantial growth since the early 20th century, reflecting their ecological and evolutionary significance. Methods: This paper provides a comprehensive bibliometric analysis of orchid-related literature published between 1902 and 2024, based on data retrieved from the Web of Science Core Collection™ (WoS). Results: The primary goal is to assess the global research landscape of orchids by identifying key authors, institutions, and journals, as well as major research themes in the field. A thorough analysis of publication trends, citation frequencies, and keyword co-occurrence networks was conducted to uncover significant research hotspots. The findings indicate that orchid research has evolved from foundational topics such as taxonomy and classification to more intricate subjects, including conservation strategies, orchid-pollinator dynamics, and the role of orchids in ecosystem functions. Additionally, biotechnology-related research is emerging as a dominant trend. This study also highlights that China has the highest publication output, while collaboration between the United States and Europe continues to grow. The co-word analysis of keywords suggests that future research is likely to continue to focus on orchid conservation, the impacts of climate change, pollination biology, and symbiotic relationships with mycorrhizal fungi. Conclusions: This review offers valuable insights for researchers and conservationists, helping to identify future research priorities and strategies for the preservation and sustainable use of orchids.

## 1. Introduction

The sustainable development of ecosystems is closely linked to the conservation and utilization of plant diversity [1,2]. Orchidaceae, one of the largest and most diverse plant families, plays a vital role in maintaining biodiversity and is potentially useful as an ecological indicator [3]. With nearly 28,000 species distributed across a wide range of ecosystems, orchids have garnered significant scientific interest due to their unique evolutionary traits, reproductive mechanisms, and ecological adaptations [4]. Despite their importance, orchids are among the most threatened plant groups, facing numerous challenges such as habitat destruction, climate change, and overexploitation [5]. Therefore, understanding the biology, ecology, and conservation of orchids is crucial for the preservation of plant diversity and the sustainability of ecosystems [6]. Over the past century, research on orchids has grown extensively, spanning multiple scientific disciplines, including evolutionary biology [7], pollination ecology [8], reproductive strategies [9], mycorrhizal interactions [10], conservation science [5], and biotechnology [11]. These studies have provided significant insights into the evolutionary success of orchids, their ecological roles, and the challenges they face in conservation.

Orchid diversity and survival are influenced by a combination of intrinsic and extrinsic factors. Intrinsic factors refer to biological characteristics that affect orchid survival and reproduction, such as specialized pollination mechanisms [12], reliance on mycorrhizal fungi for seed germination [13], and reproductive barriers like pollination limitation and inbreeding depression [14]. These factors substantially impact the reproductive success and population dynamics of orchids. Extrinsic factors, on the other hand, encompass environmental and anthropogenic threats. Nonbiological factors like climate change and habitat loss have caused significant shifts in orchid distributions and ecological interactions [15]. Biological factors, including competition with invasive species and a decline in pollinators, further exacerbate the challenges orchids face [16].

The study of orchids encompasses several major research areas. One of the primary focuses is evolutionary biology, where molecular phylogenetics has revealed patterns of diversification, hybridization, and adaptation in Orchidaceae [17]. Pollination biology and reproductive strategies represent another key research area, with studies exploring deceptive pollination, specialized pollinator interactions, and pollen limitation in different orchid species [8]. Mycorrhizal symbiosis, an essential component of orchid ecology, has been extensively studied to understand the complex nutrient exchange mechanisms between orchids and their fungal partners [18]. Conservation biology has also emerged as a critical field, addressing habitat degradation, climate change impacts, and the sustainable management of orchid populations [19]. Recent advancements in biotechnology and molecular research have facilitated in vitro propagation, genetic transformation, and secondary metabolite analysis in orchids, with implications for conservation and commercial applications [11]. Despite significant progress in orchid research, many challenges remain in understanding the complexities of orchid evolution, ecology, and conservation. Research on the global distribution of orchids, their responses to environmental changes, and their potential applications in horticulture and medicine requires further exploration. Moreover, the rapid accumulation of literature on orchids makes it difficult to comprehensively assess the development trends and emerging research frontiers in this field. Traditional review methods are time-consuming and may overlook critical knowledge gaps. However, bibliometric analysis, combined with quantitative literature analysis, provides an effective approach for identifying research trends and guiding future studies. Accordingly, this study aims to address two fundamental research questions in the field of orchid research: (1) What is the current research status of Orchidaceae, including key authors, journals, research categories, and keywords? (2) What are the potential research opportunities and future directions for scholars in the orchid research field? By applying bibliometric methods, this study seeks to provide a systematic overview of orchid research, highlight key themes and trends, and propose directions for future investigations in this rapidly evolving field.

## 2. Materials and Methods

### 2.1. Data Collection

The data for this study were obtained from the Web of Science Core Collection™ (WoS), a widely recognized and comprehensive database that provides access to high-quality, peer-reviewed scientific literature. WoS was selected due to its vast coverage across various disciplines and its ability to provide citation data, which is essential for bibliometric analysis. To ensure the reliability of the data, only English-language publications relevant to Orchidaceae were included. The search was conducted in January 2025 using the topic search term “TS = (Orchidaceae OR orchid)”, which ensured that all publications pertaining to this plant family were retrieved. The search period was not restricted to a particular date range, as the aim was to capture all publications related to Orchidaceae up until the date of the search. In order to analyze and visualize the publication trends, four distinct visualization tools were utilized: CiteSpace version 6.4.1 [20], VOSviewer version 1.6.20 [21], HistCite™ Pro version 2.1 [22], and R version 4.4.1 [23]. Each tool was selected for its specific strengths in providing insights into publication trends, key authors, institutional collaboration, and research hotspots.

### 2.2. Bibliometric Analysis

Bibliometric analysis is a systematic method used to quantitatively examine scientific publications, with the aim of identifying key research trends and phenomena [24]. Initially applied in library and information science, bibliometric techniques have since expanded to evaluate scientific progress across various fields [25]. By utilizing mathematical and statistical methods, bibliometric analysis explores the distribution, patterns, and structural regularities of the data, offering insights into the underlying science and technology trends [26]. The main objectives of the bibliometric analysis were to identify key research trends, the most influential authors and institutions, and the research topics that have gained prominence over time.

### 2.3. Impact Factor

The impact factor, first proposed by Garfield in Science, is a metric that quantifies the influence of academic journals based on the number of citations their articles receive over time [27]. It is calculated by dividing the number of citations received by articles published in a given journal during the two preceding years by the number of articles published in the same journal during the same period. In this study, the impact factor was used to assess the quality and influence of the journals that published the retrieved articles. Impact factors from the 2024 Journal Citation Reports (JCR) were applied in the analysis to determine the relative impact of journals in the Orchidaceae research domain. The JCR database provides comprehensive citation data and impact metrics, which were crucial for understanding the broader influence of different journals within the field. Journals with higher impact factors were identified as central to the orchid research community, while those with lower impact factors were considered to have a more niche or specialized focus [28]. The impact factor was also used to correlate the citation patterns of influential journals with specific research topics, helping to identify areas where significant scientific contributions have been made. This assessment of journal influence provided valuable insights into the dissemination and impact of orchid research across various sub-disciplines.

## 3. Results

### 3.1. Primary Performance of Selected Publications

**Figure 1:**
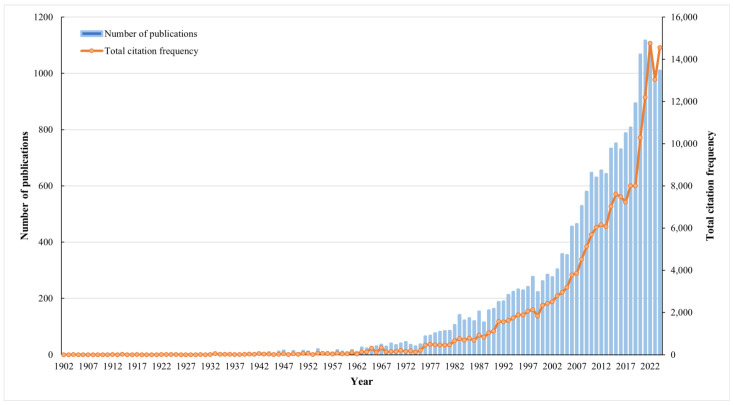
Annual trends in the number of publications and total citation frequency related to orchid research, as indexed in the Science Citation Index Expanded (WoS) from 1902 to 2024.

Figure 1 presents an overview of the publication trends in orchid-related research from 1902 to 2024. The data reveal a significant increase in annual publications, citation counts, and average references per paper. Specifically, the number of orchid-related articles grew from a single publication in 1902 to 1010 in 2024, reflecting an annual growth rate of 12.18%,which is significantly higher than the growth rate of 5.60% for all scientific literature [29]. This upward trajectory highlights the increasing academic interest in orchid studies. Furthermore, the number of citations has consistently exceeded 1000 per year for the past 36 years, with a notable increase surpassing 10,000 annually in the last five years. The average citation rate per article has risen from 9.64 in 2020 to 14.41 in 2024, approaching the global average of 15.76 for scientific publications [30]. These trends underscore a significant improvement in scholarly communication and collaboration among orchid researchers.

### 3.2. Author Collaboration and Subject Categories

**Figure 2:**
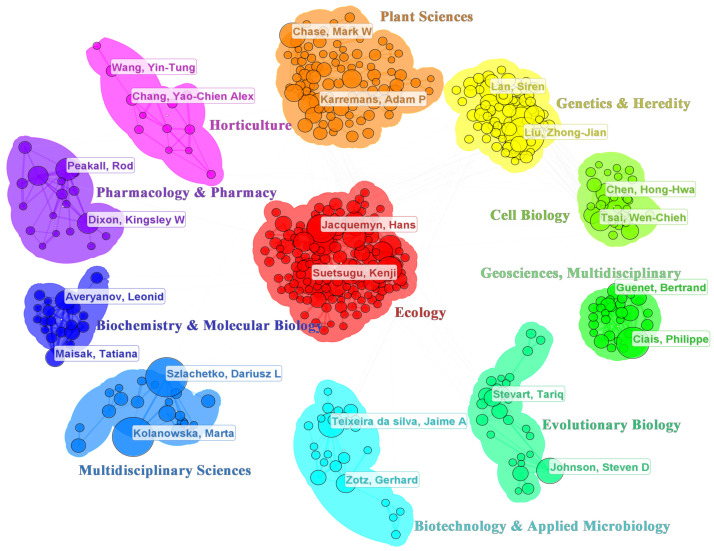
Author collaboration network, where each node represents an individual researcher. The size of the nodes corresponds to the number of publications by each author, while different cluster colors indicate distinct research topic.

The identification of core authors was conducted using Price’s Law, with the formula $M=0.749\times \sqrt{N_{max}}$, where *N_max_* represents the highest number of papers published by a single author in this field. Authors with more than ten publications (*M* = 9.85; *N_max_* = 173 obtained from WoS) were classified as core contributors. The author collaboration network (Figure 2) consists of 2514 nodes and 3791 links, indicating a well-connected research community. Prominent contributors include Marta Kolanowska (173), Dariusz L. Szlachetko (172), Zhongjian Liu (159), Kenji Suetsugu (151), and Philippe Ciais (105), who have significantly influenced orchid research. These scholars have focused on various domains such as ecology, multidisciplinary sciences, geosciences, genetics, and heredity. Additionally, Mark W. Chase has made significant contributions in plant sciences, particularly in orchid systematics and phylogenetics; Steven D. Johnson has played a critical role in evolutionary biology, especially in plant-pollinator interactions; and Wen-Chieh Tsai has contributed extensively to cell biology, focusing on orchid floral development and gene regulation. While these disciplinary classifications are based on WoS and CiteSpace statistics, they are not completely independent but rather interrelated and mutually influential.

### 3.3. Distribution of Journals, Research Institutions

From 1902 to 2024, orchid-related research was disseminated across 3308 journals. Table 1 ranks journals based on TLCS (Total Local Citation Score), a metric indicating their influence within this research domain. Among them, New Phytologist emerges as the most influential, with the highest impact factor (8.3), followed by American Journal of Botany, Botanical Journal of the Linnean Society, Annals of Botany, and Plant Systematics and Evolution. Additional journals such as Phytotaxa also contribute significantly, with a high volume of publications.

Institutional analysis was conducted using HistCite™, ranking organizations based on TLCS to determine the most influential research institutions (Table 2). The Royal Botanic Gardens, Kew, along with Chinese Academy of Sciences, University of Western Australia, University of Florida, and Australian National University ranked as the top five institutions leading orchid research.

**Figure 3:**
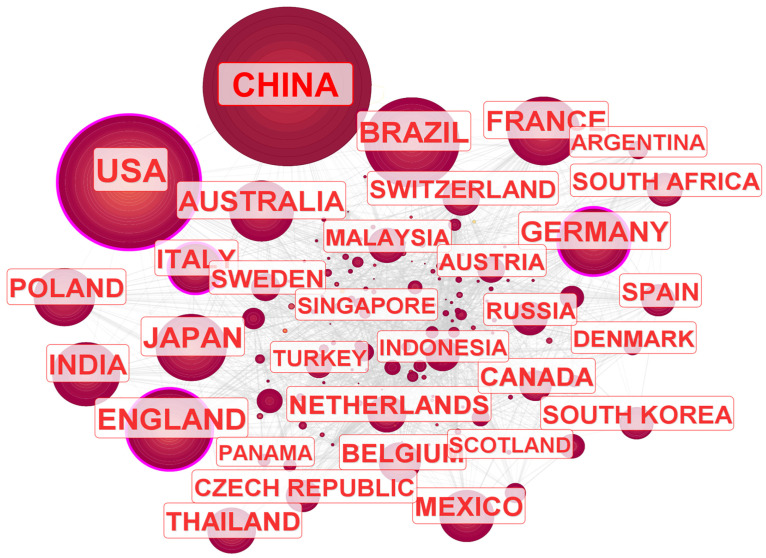
Country-level collaboration network, illustrating academic partnerships between nations. Each node represents a country, with critical nodes highlighted by a purple ring. Lines connecting nodes signify cooperative research relationships.

Global academic collaboration was examined through a country network analysis using CiteSpace. Betweenness centrality [31], a metric for measuring a country’s influence within the network, was calculated. A node surrounded by a purple ring indicates a highly influential country (Figure 3). China contributed 3424 publications (16.47% of total papers), equaling the United States (3424 papers). However, while China exhibited a high volume of publications, its betweenness centrality remained relatively low. In contrast, the United States demonstrated both a high article count and strong academic influence, reflected in its betweenness centrality (0.19). A regional analysis showed that European countries, including the United Kingdom, Germany, and Italy, collaborated closely and exhibited strong influence in orchid research.

To provide a broader geographical perspective, we categorized the data by continents, recognizing six major landmasses: Asia, Europe, North America, South America, Africa, and Oceania (Table 3). The analysis revealed that Europe (9224 papers) and Asia (8089 papers) contributed the highest number of publications, whereas North America (4695 papers) and Oceania (1083 papers) demonstrated strong academic influence despite lower publication volumes. A regional analysis showed that European countries, including the United Kingdom, Germany, and Italy, collaborated closely and exhibited strong influence in orchid research, while China, Japan, and India led the research efforts in Asia. North America was primarily represented by the United States, Canada, and Mexico, whereas Australia and New Zealand dominated Oceania’s contributions.

**Figure 4:**
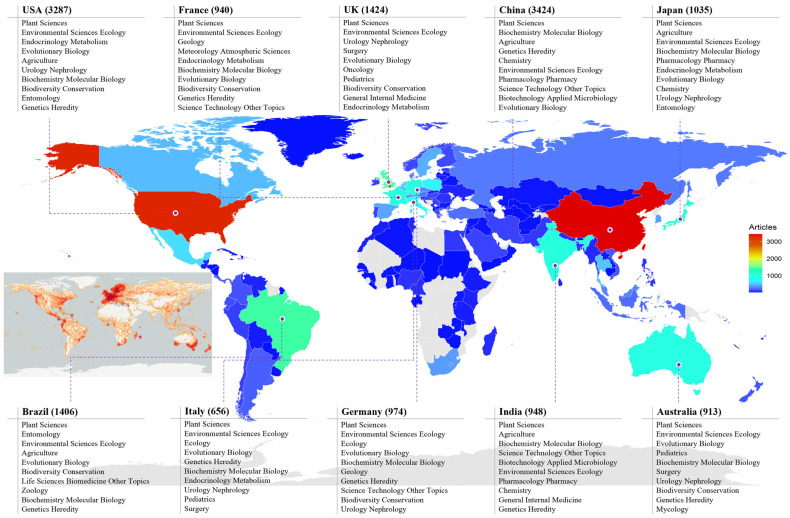
Global distribution of publications on orchid research, displaying the top 10 research areas in the 10 most highly contributing countries, based on data retrieved from the Web of Science Core Collection. The red pentagram indicates the earliest reported region of orchid studies: London. The smaller inset map, located in the middle-left side of the figure, is sourced from the Global Biodiversity Information Facility (GBIF) [32] and highlights the global distribution of orchid species. The map uses color-coded data points, with the orange-red areas indicating regions of high species density.

Figure 4 presents the top 10 countries’ research directions, revealing that while all major contributors focused on Plant Sciences and Environmental Sciences Ecology, some countries specialized in distinct fields—Oncology in the UK and Mycology in Australia (Figure 4). Moreover, a comparison of research output with global orchid diversity distribution suggests that the highest concentration of orchids is observed in Europe, parts of North America, and certain regions in Asia and Oceania. In contrast, areas like Africa, South America, and Southeast Asia, despite being biodiversity hotspots, show a comparatively lower density in this distribution map. This highlights potential gaps in research coverage, suggesting the need for increased studies in biodiversity-rich but underrepresented regions.

### 3.4. Time-Series Analysis of High-Frequency Keywords and Emerging Topics

**Figure 5:**
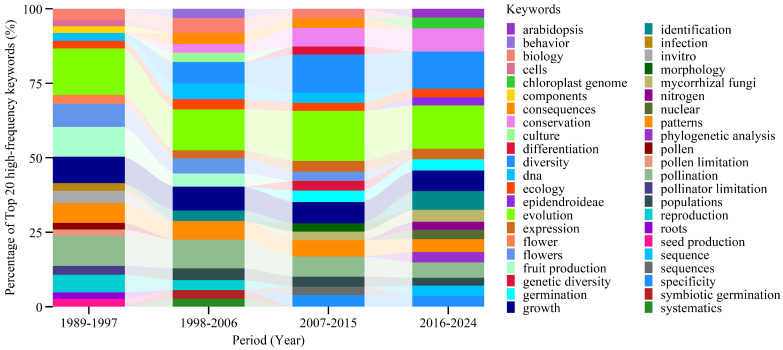
Temporal trends of high—frequency keywords from 1989 to 2024.

Orchid research from 1902 to 1988 primarily focused on cultivation and morphological classification [33,34]. A shift occurred in 1989, marking the emergence of industrial applications of orchids [35]; Figure 5 illustrates the nine-year keyword evolution cycles (1989–2024) tracking the composition and ranking of the top 20 keywords per cycle (excluding generic terms such as “Orchidaceae plant” and “size”). Throughout the research period, “evolution” remained a dominant topic. In recent years, “conservation” and “identification” have replaced earlier terms such as “patterns” and “fruit production” in the top six keywords. Additionally, “growth” and “pollination” have consistently remained among the most frequently used terms. Notably, “systematics” and “symbiotic germination” experienced a surge in research interest after 1998, reflecting the growing impact of molecular techniques in orchid studies. Furthermore, “diversity” gained prominence, highlighting an increased focus on biodiversity conservation. The advancement of computer-assisted research methodologies has enabled scholars to analyze endangered plants more effectively, leading to breakthroughs in conservation biology and wildlife management. Moreover, the 1990s marked a revolution in orchid research with the widespread adoption of DNA-based phylogenetics and molecular ecology methods. Techniques such as sequencing of rbcL, matK, and ITS regions enabled more precise classification and evolutionary studies [36].

### 3.5. Evolution of Research Hotspots

**Figure 6:**
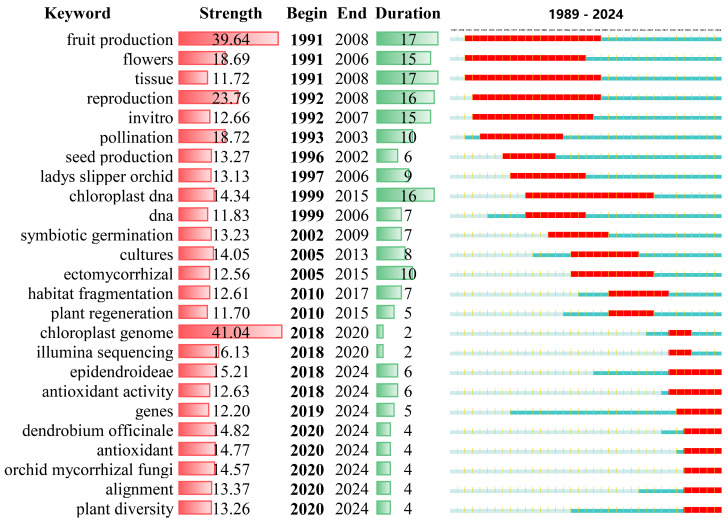
Top 25 keywords detected by CiteSpace based on burst detection, displaying their strength and duration. The strength values correspond to the frequency of citations associated with each keyword.

Burst keywords typically represent emerging research trends, rising topics, or sudden surges in attention within a particular field. These keywords frequently emerge within a short time frame and often indicate key areas of research or innovative breakthroughs that capture academic or industry focus during a specific period. Therefore, we applied CiteSpace to analyze burst keywords in orchid research. However, the number of articles prior to 1989 was insufficient to meet the basic requirements for statistical analysis. As a result, we focused our analysis on research published since 1989. As shown in Figure 6, the effective statistical analysis reveals that the evolution of research can be divided into three distinct stages:

In the first stage (1991–2004), there were more explosive keywords, and many keywords with high emergence intensity. After 1991, the keywords that quickly became the research hotspots of orchids included: “fruit production”, “flowers”, “reproduction”, and “pollination”. At this stage, the “reproduction” keyword received high emergence intensity, indicating a strong focus on the mechanisms behind orchid reproduction. Research during this period emphasized the diversity of reproductive strategies in orchids, including their reliance on specific pollinators and the role of pollination ecology in their survival. Pollination biology in orchids, especially their relationships with insects like bees and moths, became a central topic [37]. Additionally, research on the “flower” keyword rose during this period, focusing on floral morphology, fragrance, and color patterns, which are critical for attracting pollinators. Orchids were studied for their diverse floral adaptations, such as mimicry and specific pollinator attractants, which play a crucial role in their reproductive success [38].

In the second stage (2005–2017), although there were fewer explosive keywords, many keywords experienced long outbreak periods, such as the keyword “ectomycorrhizal”. After 2005, numerous studies focused on ectomycorrhizal fungi and their symbiotic relationships with orchid roots. This relationship is vital for nutrient uptake, especially in nutrient-poor soils, and plays a critical role in the establishment and survival of many plant species, particularly in forest and terrestrial ecosystems [39]. However, research on orchids specifically has shifted toward understanding the interactions between specific ectomycorrhizal fungi and orchid species. These fungi are not only essential for nutrient absorption but also facilitate seed germination and early seedling development in orchids, which is a key aspect of their reproductive strategy. In parallel, the keywords “habitat fragmentation” and “plant regeneration” continued to show explosive patterns for 8 years and 6 years, respectively. The sustained interest in “habitat fragmentation” was mainly due to its widespread recognition as a major threat to biodiversity. Habitat fragmentation leads to the formation of small, isolated populations, reducing genetic diversity and altering ecological dynamics [40]. This trend is particularly pertinent to orchids, which often have specific habitat requirements and face heightened risks from fragmentation due to their dependence on particular ecological conditions, such as pollinators and symbiotic relationships with fungi. Studies during this period focused on how ecological restoration measures could mitigate the negative impacts of habitat fragmentation and enhance orchid population viability. Similarly, “plant regeneration” emerged as a critical keyword because the ability of orchids to regenerate, whether through seed germination, vegetative growth, or clonal reproduction, is central to ecosystem recovery and resilience. Researchers have particularly focused on how fragmentation and environmental stressors affect regeneration processes, especially in endangered or declining orchid species [41]. Habitat fragmentation often disrupts natural regeneration dynamics by limiting seed dispersal, reducing genetic diversity, and altering soil microbial communities, ultimately hindering population recovery and biodiversity conservation. Consequently, ongoing studies aim to identify ecological restoration strategies that can mitigate these adverse effects and enhance plant regeneration, particularly in degraded environments. Given the increasing threats posed by habitat loss and fragmentation, research on the interplay between ecological disturbances and plant regeneration has gained significant attention. Understanding how fragmentation affects orchid regeneration processes is crucial for developing effective conservation strategies that mitigate its impacts and support long-term plant biodiversity [42].

In the third stage (2018 to present), a significant number of burst keywords emerged, with several not only exhibiting high emergence intensity but also sustained periods of attention. Notably, the keyword “chloroplast genome” displayed the highest strength value of 41.04, with a burst period from 2018 to 2020. This surge in interest reflects the growing recognition of the chloroplast genome as a key area in orchid genomics, as it provides valuable insights into the evolutionary relationships, genetic diversity, and adaptive traits of orchids. Meanwhile, Illumina sequencing technology, with its high-throughput and accuracy, has revolutionized plant genomics, enabling researchers to uncover complex genetic variations and functional traits in orchids [43]. This technology has significantly advanced our understanding of orchid species at the molecular level [44]. Concurrently, the growing emphasis on “antioxidant activity” highlights its vital role in plant defense mechanisms and potential health benefits, particularly in relation to medicinal plants. Keywords such as “*Dendrobium officinale*” and “antioxidant” have received continuous attention, as studies delve into the bioactive compounds in this plant and its therapeutic potential [45]. In addition, the application of biotechnologies focused on “epidendroideae” and “plant diversity” in the field of orchids will continue to attract attention. Researchers are increasingly exploring the conservation and propagation of endangered orchid species within the Epidendroideae subfamily, driven by both ecological concerns and their commercial value [4]. The commercial value of orchids has extended beyond ornamental purposes to encompass pharmaceuticals, food products, natural flavorings, biofunctional materials, and ecological restoration. Notably, species such as *D. officinale*, *Gastrodia elata*, *Arundina graminifolia*, and *Bletilla striata* are widely utilized in traditional medicine due to their antioxidant, neuroprotective, and wound-healing properties [46,47], while *Vanilla planifolia* remains a cornerstone of the natural flavoring industry [48]. Furthermore, emerging applications of *Anoectochilus roxburghii* in functional foods and *Cymbidium* spp. in edible flower production highlight the diversification of orchid-based industries [49,50,51]. The integration of cutting-edge techniques such as genetic engineering, tissue culture, and synthetic biology will be vital for preserving orchid diversity and improving the sustainability of orchid industries. Recent advancements in biotechnological interventions, including genomic sequencing and transgenic approaches, are enhancing the large-scale propagation and conservation of economically significant orchids, thereby accelerating their industrialization [52].

### 3.6. Research Field Classification

**Figure 7:**
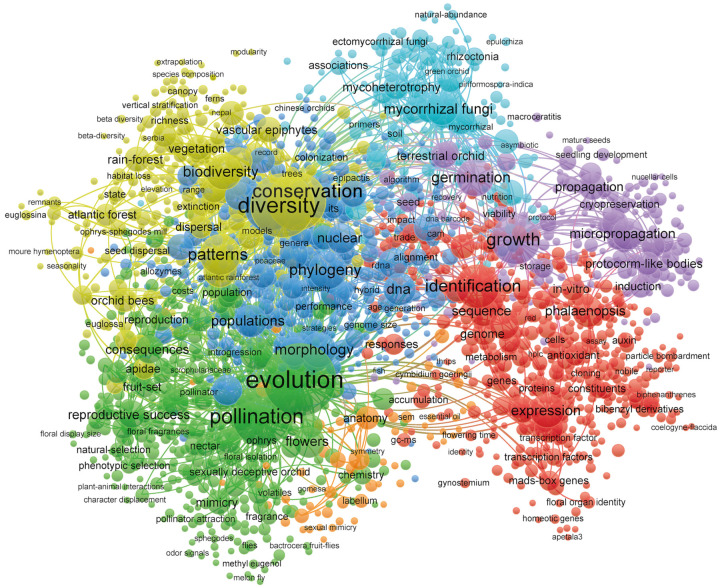
Keyword co-occurrence network illustrating research focus and interconnections. Each cluster represents a distinct thematic area of orchid research. The clusters are color-coded, where each color corresponds to a specific research theme. Green: Evolution and pollination mechanisms; Yellow: Orchid conservation and biodiversity; Purple: In vitro propagation and germination; Red: Molecular mechanisms and biotechnology; Blue: Phylogenetics and taxonomy; Cyan: Mycorrhizal symbiosis and nutrient acquisition; Orange: Morphological and anatomical features.

A co-occurrence analysis was conducted to systematically categorize orchid research into seven thematic clusters, as shown in Figure 7. This approach, using VOSviewer, allowed for the identification of key areas of focus in the orchid research landscape by analyzing the co-occurrence of keywords across the literature.

#### 3.6.1. The Impact of Evolutionary and Pollination Mechanisms in Orchidaceae Research

The green cluster in Figure 7 represents this research field, where a major theme is the investigation of evolutionary processes such as adaptation, speciation, and radiation, reflected by keywords like “evolution”, “pollination”, and “reproductive success”. These topics illustrate the complexity of orchid reproduction and the influence of pollinators on evolutionary trajectories. Many orchids, such as those in the Ophrys genus [53], have evolved sexually deceptive strategies, whereby their flowers mimic the visual and chemical cues of female insects to attract male pollinators, a mechanism known as “deceptive pollination” [7]. This pollination strategy has significant evolutionary consequences, driving selection on floral traits such as size, fragrance, and shape while also shaping pollinator behavior and specialization [54].

A key distinction in orchid pollination strategies lies between food-deceptive and rewarding mechanisms. Food-deceptive orchids attract pollinators by mimicking food rewards, such as nectar, but provide no nutritional benefit. While this leads to high visitation rates, it also increases the risk of geitonogamy, reducing genetic diversity and reproductive success through inbreeding [55]. Conversely, rewarding orchids offer actual food rewards, encouraging cross-pollination and promoting genetic diversity. However, they face challenges in balancing pollinator attraction with avoiding self-pollination, especially in dense floral populations [7]. Both strategies are critical for orchid conservation, as effective pollination is essential for maintaining genetic diversity, particularly in the face of habitat degradation and declining pollinator populations.

A particularly interesting discovery is the role of “pollinator-mediated selection” which has been shown to shape key floral traits like pigmentation, size, and scent production in response to specific pollinator preferences [56]. Another crucial concept in this field is pollination limitation, where a scarcity of pollinator visits directly constrains reproductive success, thereby impacting orchid population dynamics and species distribution patterns [57]. Understanding these reproductive strategies is fundamental to orchid conservation, particularly as many species face significant threats from habitat destruction, declining pollinator populations, and climate change [58].

#### 3.6.2. Orchid Conservation and Biodiversity Patterns in Changing Environments

The conservation of orchids and the biodiversity patterns observed across various ecosystems, particularly in tropical and subtropical regions, are represented within the yellow cluster in Figure 7. This research domain focuses on the interaction between orchid species and their surrounding environments, emphasizing ecological processes such as habitat fragmentation, climate change, and human-induced disturbances. Key terms like “diversity”, “conservation”, “biodiversity”, and “ecology” highlight the importance of understanding orchid distribution and genetic diversity in response to environmental changes. Genetic diversity within orchid populations is crucial for their long-term survival and adaptability, as it provides the raw material for evolutionary processes and resilience to environmental stressors. Factors such as “habitat fragmentation”, “seed dispersal”, and “climate change” play critical roles in shaping orchid populations and their ability to adapt to increasingly disturbed habitats [59]. Moreover, keywords such as “orchid bees”, “Euglossini”, and “pollinators” underscore the role of pollinator interactions in maintaining orchid genetic diversity and reproductive success. Since many orchids depend on specific pollinators, particularly Euglossine bees, their survival is tightly linked to pollinator availability, making them vulnerable to environmental changes, deforestation, and habitat loss. Additionally, this research cluster integrates predictive modeling techniques such as “species distribution models” and “biodiversity conservation” to anticipate future distribution shifts and inform conservation strategies. Several studies have highlighted the need for effective restoration and management practices to protect both orchids and their associated ecosystems [60]. Overall, the ongoing research in this domain aims to unravel the intricate relationships among ecological, geographical, and anthropogenic factors shaping orchid species distributions, with a strong emphasis on safeguarding rare and endangered species in biodiversity hotspots. The application of advanced ecological modeling and field surveys will continue to play a crucial role in predicting orchid population responses to environmental changes and guiding conservation policies.

#### 3.6.3. In Vitro Propagation and Germination of Orchids

The purple cluster in Figure 7 represents the research area focusing on in vitro propagation, germination, and plant regeneration in orchids. Keywords such as “germination”, “micropropagation”, and “growth” reflect the significance of developing effective propagation techniques to ensure species survival, especially for orchids with restricted natural habitats. The application of in vitro germination methods, including asymbiotic germination and tissue culture, has enabled researchers to circumvent the challenges posed by orchid seed dormancy and low germination rates, particularly in terrestrial orchids [61]. Techniques such as protocorm-like bodies (PLBs) formation and somatic embryogenesis have been widely used for large-scale propagation, supporting both conservation and commercial breeding programs [62]. Furthermore, cryopreservation technologies have been developed to store orchid seeds and germplasm long-term, offering a safeguard against extinction for vulnerable species [63]. Optimization of culture media composition, light exposure, and plant growth regulators (such as cytokinins and auxins) has been instrumental in enhancing the efficiency of propagation methods [64]. As research in this domain advances, in vitro culture techniques will remain an essential tool for orchid conservation, offering a sustainable approach for restoring threatened species, maintaining genetic diversity, and facilitating reintroduction programs in degraded environments [65].

#### 3.6.4. Molecular Mechanisms and Biotechnology in Orchid Research

The red clusters in Figure 7 represent key research areas focusing on molecular identification, gene expression, and stress responses in orchids. Keywords such as “identification”, “expression”, “gene”, and “protein” reflect an increasing interest in the genetic and molecular foundations of orchid physiology, particularly in response to environmental stressors. Commonly studied orchids, such as Phalaenopsis and Dendrobium, have been the focus of research into gene expression regulation, secondary metabolite biosynthesis, and plant response mechanisms to abiotic and biotic stresses, including temperature fluctuations, drought, and light conditions. Studies have emphasized the roles of MADS-box genes, transcription factors, and key metabolic pathways such as crassulacean acid metabolism (CAM) and photosynthesis, which are crucial for orchids’ adaptation to extreme environments. Although CAM is a well-established photosynthetic pathway, its specific role in orchids, such as *Taeniophyllum* and *Cymbidium*, has been more clearly defined in recent years, largely due to advances in sequencing technologies. In *Taeniophyllum* [66], CAM is essential for the adaptation of its aerial root to epiphytic conditions, while in *Cymbidium* [51], CAM enhances water-use efficiency, helping these orchids thrive in fluctuating environments. In addition, biotechnology applications, including in vitro propagation, genetic transformation, and RNA sequencing (RNA-seq), have facilitated rapid advancements in orchid breeding and conservation. These technologies not only support conservation initiatives but also contribute to improving disease resistance, flowering control, and the production of medicinal compounds [51].

#### 3.6.5. Phylogenetics and Taxonomy in Orchid Research

The blue clusters in Figure 7 encompass research focusing on phylogenetics, taxonomy, and genetic diversity in orchids. The study of orchid phylogeny has been largely driven by the use of molecular techniques, with keywords such as “phylogeny”, “taxonomy”, “morphology”, “speciation”, and “genetic diversity” indicating a strong focus on species classification and evolutionary relationships. Researchers have relied on molecular markers such as internal transcribed spacers (ITS), chloroplast DNA, and nuclear ribosomal DNA to reconstruct phylogenetic trees, infer species diversification events, and assess hybridization patterns [67]. Integrating morphological traits with genetic data has significantly refined orchid systematics, especially within major subfamilies like Epidendroideae, Orchidoideae, and Vanilloideae.

Recent studies have provided significant insights into the molecular evolution of orchids, particularly through the application of genomic techniques. Chloroplast genome analyses have elucidated the phylogenetic relationships within key genera, such as *Cymbidium*, offering new perspectives on their ecological niche adaptations [68]. The draft genome of *Apostasia shenzhenica* has been pivotal in uncovering the evolutionary origins of orchids, shedding light on early diversification patterns within the family [69]. Genomic studies on *Pleione* species from the Himalayas have underscored the influence of climatic factors in driving species diversification and shaping their distribution [70]. Additionally, molecular tools have advanced our understanding of gene flow and hybridization dynamics in genera like *Epidendrum*, revealing complex patterns of evolutionary divergence and adaptation [71]. As a representative of one of the earliest diverging lineages in the Orchidaceae, recent molecular investigations into *Angraecum* (Darwin’s orchid) have highlighted the convergent evolution of oxime biosynthesis, illustrating the intricate biochemical and ecological adaptations that enable orchids to thrive in a variety of environmental contexts [69].

#### 3.6.6. Mycorrhizal Symbiosis and Nutrient Acquisition in Orchids

The cyan clusters in Figure 7 emphasize the critical role of mycorrhizal fungi in orchid ecology, seed germination, and nutrient uptake. Orchids form specialized mycorrhizal associations, facilitating the acquisition of nitrogen, carbon, and phosphorus from the environment essential for survival in nutrient-deficient habitats [72]. These interactions are particularly vital during early seedling development, where mycorrhizal fungi enable successful establishment in ecosystems that would otherwise be inhospitable to orchid seeds. Since orchid seeds are very small and lack nutritional reserves, they produce a large number of seeds compared to many other plants. However, this strategy comes at a cost: during early stages, seedlings are entirely dependent on fungal-derived nutrients, making them highly vulnerable. Different fungal groups, such as *Rhizoctonia*, *Tulasnella*, and *Ceratobasidium*, display varying levels of host specificity with orchids, influencing nutrient exchange and seedling survival rates [73]. The evolution of mycoheterotrophic and mixotrophic nutrient strategies in orchids further demonstrates their adaptive flexibility, with some species fully dependent on fungal-derived carbon sources [74]. One well-documented example of orchids forming highly specific relationships with mycorrhizal fungi is *Cypripedium guttatum* (the spotted lady’s slipper orchid). This species forms a highly specific association with a group of fungi in the genus *Tulasnella*. The mutualistic relationship between *C. guttatum* and *Tulasnella* fungi is so specific that the orchid can only germinate and establish successfully in the presence of these particular fungi. This specificity has been shown to limit the distribution of the orchid to certain habitats where *Tulasnella* is abundant, thus restricting its ecological range [75]. Advances in high-throughput sequencing and stable isotope analysis have provided deeper insights into fungal community dynamics, mycorrhizal network interactions, and their ecological significance in orchid diversity and distribution [76]. For instance, a study on *Anacamptis morio* investigated temporal shifts in mycorrhizal fungal assemblages and stable isotope composition over different phenological stages [77]. Using high-throughput sequencing, the study revealed significant seasonal variations in fungal partners, with *Tulasnella* dominating in autumn and winter, *Pezizaceae* in early spring, and *Ceratobasidium* in late spring and summer. Despite these shifts, stable isotope analysis indicated relatively stable nitrogen and carbon isotopic compositions in the orchid leaves, suggesting a consistent nutrient acquisition strategy irrespective of fungal turnover. These findings underscore the dynamic nature of orchid-mycorrhizal interactions while highlighting the stable isotope approach as a powerful tool to elucidate nutritional dynamics in orchid symbioses. Understanding these relationships is essential for conservation efforts, as orchid-associated fungi play a crucial role in habitat restoration and species recovery programs.

#### 3.6.7. Morphological and Anatomical Features of Orchids

The orange clusters in Figure 7 highlight research on the morphology and anatomy of orchids, particularly their structural adaptations for pollination and reproductive success. Studies on micromorphology and ultrastructure, focusing on the labellum, nectary, and gynostemium, have revealed specialized floral structures that support pollination and mimicry strategies [78]. Features such as pseudopollen, oil glands, and extrafloral nectaries play crucial roles in attracting pollinators, with sexual mimicry being a key strategy for reproductive isolation and genetic diversity. Advanced imaging techniques, including scanning electron microscopy (SEM) and transmission electron microscopy (TEM), have provided insights into trichome arrangement, papillae formation, and nectar secretion pathways, which influence pollination success [79]. Additionally, research on cytoskeletal components and mitotic activity during floral development has enhanced our understanding of petal differentiation and reproductive organ formation [80]. These morphological adaptations contribute to orchids’ ecological success, allowing them to thrive in diverse environments by interacting with specific pollinators such as ants, bees, and butterflies. Future studies integrating molecular genetics and ecological modeling will further clarify the evolutionary drivers of floral diversity and inform conservation efforts for endangered orchid species.

## 4. Discussion

### 4.1. Key Issues in the Orchid Field

Orchidaceae, as one of the most diverse and ecologically significant plant families, has been extensively studied in various disciplines, including evolution, taxonomy, pollination biology, reproductive ecology, mycorrhizal interactions, conservation biology, and biotechnology. According to bibliometric keyword analysis, the most researched topics in orchids can be classified into five main areas: phylogenetics and evolutionary biology, reproductive mechanisms and pollination, ecological adaptations, conservation strategies, and biotechnology applications. Phylogenetic research has significantly contributed to understanding orchid diversification, with frequent references to “phylogenetic analysis” and “molecular phylogenetics”. Pollination biology remains a dominant research theme, as indicated by high-frequency keywords such as “pollination”, “reproductive isolation”, and “sexual deception”, reflecting the importance of specialized pollinator interactions. Mycorrhizal symbiosis is another critical area, with keywords such as “mycorrhiza”, “symbiotic germination”, and “fungi” highlighting the dependence of orchids on fungal partners for seed germination and nutrient acquisition [81]. In addition, research on conservation strategies, including in situ and ex situ conservation, is gaining prominence due to habitat destruction and climate change threats [82]. Finally, advancements in biotechnology, genome sequencing, and secondary metabolite studies have enabled deeper insights into orchid genetics, propagation, and medicinal applications [83].

The factors influencing orchid diversity and survival can be categorized into intrinsic biological limitations and extrinsic environmental pressures. Intrinsic factors include low genetic diversity, specialized pollination mechanisms, reproductive barriers, and mycorrhizal dependency, which can restrict orchid population growth and regeneration. The keyword “pollen limitation” appears frequently in orchid studies, emphasizing the challenges posed by pollinator specialization and pollinator declines [84]. Inbreeding depression and seed dormancy are also recurring issues that affect reproductive success. On the other hand, extrinsic threats to orchids include climate change, habitat fragmentation, and human disturbances [85]. Keywords such as “climate change”, “temperature”, and “habitat fragmentation” highlight the increasing concern over environmental changes affecting orchid distributions. Anthropogenic activities such as deforestation, overharvesting, and land-use changes have led to drastic population declines in many orchid species. The frequency of terms like “protected areas” and “conservation genetics” indicates that researchers are focusing on mitigating these threats through conservation planning and genetic diversity assessments [86].

With growing threats to orchid populations, conservation strategies and biotechnology applications have become essential research areas. Keywords such as “ex situ conservation”, “reintroduction”, and “seed banking” reflect efforts to protect orchids outside their natural habitats [87]. In situ conservation strategies involve protecting orchids within their native ecosystems, often through national parks, botanical reserves, and habitat restoration projects. Biotechnology has also played a crucial role in orchid conservation and sustainable utilization. High-frequency keywords such as “in vitro propagation”, “somatic embryogenesis”, and “DNA marker” indicate that researchers are actively exploring methods to enhance orchid propagation, genetic diversity, and secondary metabolite production [88]. Genetic engineering and synthetic biology are emerging trends, with studies focusing on transcriptomics, genome editing, and metabolic engineering to improve orchid conservation and commercial applications. Additionally, computational approaches, including GIS-based species distribution modeling and ecological monitoring tools, are becoming valuable for predicting orchid population dynamics and planning conservation strategies [89].

### 4.2. Future Trend Analysis

Based on burst detection analysis and keyword trend analysis in CiteSpace, several emerging research directions are expected to shape the future of orchid studies. Phylogenomic research will continue to refine orchid classification, integrating whole-genome sequencing, transcriptomics, and comparative genomics to resolve taxonomic uncertainties [4]. With the increasing availability of high-throughput sequencing, studies focusing on phylogenetic relationships, molecular evolution, and genome-wide association studies (GWAS) will provide deeper insights into orchid diversification and adaptation mechanisms [90]. The increasing research focus on the chloroplast genome, indicated by its high emergence intensity in bibliometric analysis, suggests that future studies will emphasize understanding chloroplast function in orchid adaptation, photosynthesis efficiency, and species differentiation [91].

Climate change and ecological modeling will become increasingly important as orchids are highly sensitive to environmental changes. The presence of keywords such as “climate change”, “temperature”, and “habitat fragmentation” reflects growing concerns about the impact of global warming on orchid distributions and reproductive success [92]. Species distribution models (SDMs) and ecological niche modeling (ENM) have been widely used to predict shifts in orchid habitats under different climate scenarios. Future studies will likely integrate remote sensing, GIS technology, and big data analytics to enhance the accuracy of these predictive models [93]. Additionally, research on adaptive traits, phenotypic plasticity, and epigenetic regulation will help assess how orchids respond to climate variations over long evolutionary timescales [94].

Pollination biology and pollinator conservation will remain a critical focus, particularly in light of global pollinator decline. Keywords such as “pollination limitation”, “reproductive isolation”, and “pollinator-mediated selection” suggest an increasing interest in understanding orchid-pollinator interactions [95]. The role of specialized pollinators, including bees (Euglossini), moths (Sphingidae), and flies (Diptera), in orchid reproduction will continue to be a significant research area. Future studies will likely explore the influence of habitat fragmentation on pollination networks, the role of chemical cues and floral scents in pollinator attraction, and the impact of pollinator decline on orchid reproductive success [96].

The importance of mycorrhizal associations and microbial ecology in orchid survival and adaptation is expected to expand. The presence of keywords such as “mycorrhiza”, “symbiotic germination”, and “fungal networks” suggests that mycorrhizal research will remain a prominent area of study [97]. Recent advances in metagenomics and environmental DNA (eDNA) sequencing have provided new insights into the diversity and functional roles of orchid mycorrhizal fungi [98]. Future research is expected to explore the specificity of orchid–fungal relationships, investigate the impact of soil microbiomes on seedling establishment, and develop fungal-assisted restoration techniques for endangered orchid species.

Advances in biotechnology and conservation genetics will play a crucial role in the sustainable utilization and conservation of orchids. Keywords such as “in vitro propagation”, “somatic embryogenesis”, “DNA marker”, and “reintroduction” suggest that tissue culture and genetic conservation techniques will remain key areas of research [99]. Emerging biotechnological applications include genome editing (CRISPR-Cas9), synthetic biology, and metabolic engineering to enhance secondary metabolite production in medicinal orchids [100]. Additionally, DNA barcoding, molecular markers, and population genomics will be increasingly used to assess genetic diversity, identify conservation priorities, and develop breeding programs for rare and endangered orchid species [101].

Finally, computational approaches and big data applications in orchid research will continue to evolve, revolutionizing data analysis and conservation planning. The increasing use of artificial intelligence (AI), machine learning, and ecological informatics in orchid studies is expected to enhance species identification, population viability analysis, and conservation decision-making [102]. Keywords such as “software”, “GIS”, and “3S technology (GIS, RS, GPS)” suggest that spatial modeling and geospatial analysis will be increasingly applied in species distribution mapping, habitat suitability assessments, and conservation strategy optimization [103]. As data-sharing platforms and open-access databases expand, collaborative research networks and interdisciplinary approaches will become essential for advancing orchid science and conservation.

## 5. Conclusions

With rapid scientific advancements and increasing ecological concerns, Orchidaceae research has evolved into a multidisciplinary field encompassing evolutionary biology, pollination ecology, reproductive strategies, mycorrhizal interactions, conservation biology, and biotechnology. The bibliometric analysis of orchid-related literature reveals that major research themes include phylogenetics and evolutionary processes, specialized pollination mechanisms, seed germination and mycorrhizal associations, ecological adaptations, conservation strategies, and applied biotechnology. Phylogenetic studies, driven by molecular techniques, have significantly refined orchid classification, while pollination biology has deepened our understanding of deceptive and specialized pollination strategies. Orchid-fungal symbioses remain crucial to species survival, with research expanding on fungal specificity and ecological dependencies. Conservation research has gained prominence due to the increasing threats posed by climate change, habitat loss, and declining pollinator populations, emphasizing both in situ and ex situ conservation approaches. Additionally, biotechnology has played a transformative role in orchid research, with advancements in genome sequencing, transcriptomics, tissue culture, and secondary metabolite studies, particularly in economically significant orchids such as *D. officinale*. The analysis further highlights that China, Italy, and the United States are leading contributors to orchid research, with strong international collaborations fostering scientific progress.

Future research on orchids will likely focus on phylogenomics, climate resilience, pollination ecology, microbial interactions, conservation genomics, and computational modeling. The ongoing impact of climate change necessitates ecological modeling to predict orchid responses and develop adaptive conservation strategies. The decline of pollinators will drive further investigations into pollination networks and reproductive success in orchids. Advances in metagenomics and transcriptomics will enhance our understanding of mycorrhizal interactions and their role in orchid establishment. Additionally, biotechnological innovations such as gene editing, synthetic biology, and metabolomics will be crucial for both conservation and commercial applications. The integration of GIS, remote sensing, machine learning, and big data analytics will provide new insights into orchid distribution, adaptation, and conservation planning. This study offers a comprehensive overview of current trends and future research directions, providing valuable guidance for researchers aiming to advance orchid science and conservation efforts in the coming decades.

## Figures and Tables

**Table 1:** Characteristics of the top 10 journals from 1902 to 2024.

No.	Journal Name	TLCS	TGCS	Records
1	New Phytologist	8030	17,854	233
2	American Journal of Botany	7370	14,107	316
3	Botanical Journal of The Linnean Society	5895	8676	338
4	Annals of Botany	5677	9846	258
5	Plant Systematics and Evolution	3839	6299	270
6	Molecular Ecology	2670	4455	70
7	Phytochemistry	2524	6911	171
8	Phytotaxa	2302	2927	724
9	Evolution	1794	3538	49
10	Scientia Horticulturae	1709	3412	164

**Table 2:** Top 10 influential institutions from 1902 to 2024.

No.	Institution	TLCS	TGCS	Records	Country
1	Royal Botanic Gardens, Kew	7223	15,371	387	UK
2	Chinese Academy of Sciences	5318	15,406	827	China
3	University of Western Australia	4775	10,130	180	Australia
4	University of Florida	3400	7158	257	USA
5	Australian National University	2874	5711	159	Australia
6	University of Naples Federico II	2562	4201	133	Italy
7	University of Puerto Rico	2282	3522	96	USA
8	Academy Sinica	2174	5082	170	China
9	University of Bayreuth	2121	3820	69	Germany
10	University of KwaZulu Natal	2096	5696	144	SA

**Table 3:** Global orchid research distribution across six continents.

No.	Continent	Total Publications	Max Betweenness Centrality	Major Collaborating Countries
1	Asia	8089	0.07	China, Japan, India
2	Europe	9224	0.17	England, Germany, France
3	North America	4695	0.19	USA, Mexico, Canada
4	Oceania	1083	0.09	Australia, New Zealand, New Caledonia
5	South America	2070	0.02	Brazil, Argentina, Colombia
6	Africa	747	0.05	South Africa, Egypt, Nigeria

## Data Availability

The original contributions presented in the study are included in the article, further inquiries can be directed to the corresponding author.
